# Rare Dihydropyrimidine Dehydrogenase Variants and Toxicity by Floropyrimidines: A Case Report

**DOI:** 10.3389/fonc.2019.00139

**Published:** 2019-03-11

**Authors:** Raffaele Palmirotta, Domenica Lovero, Hervé Delacour, Audrey Le Roy, Serge Cremades, Franco Silvestris

**Affiliations:** ^1^Section of Clinical and Molecular Oncology, Department of Biomedical Sciences and Human Oncology, Università degli Studi di Bari, Bari, Italy; ^2^Department of Biology, Military Training Hospital Begin, Saint Mandé, France; ^3^Val-de-Grâce Military School, Paris, France; ^4^Department of Oncology, Military Training Hospital Begin, Saint Mandé, France

**Keywords:** pharmacogenomics, capecitabine, fluoropyrimidine, dihydropyrimidine dehydrogenase, gene variation, toxicity

## Abstract

Variations in the activity, up to absolute deficiency, of the enzyme dihydropyrimidine dehydrogenase (DPD), result in the occurrence of adverse reactions to chemotherapy, and have been included among the pharmacogenetic factors underlying inter-individual variability in response to fluoropyrimidines. The study of single-nucleotide polymorphisms of the *DPYD* gene, which encodes the DPD enzyme, is one of the main parameters capable of predicting reduced enzymatic activity and the consequent influence on fluoropyrimidine treatment, in terms of reduction of both adverse reactions and therapeutic efficacy in disease control. In this paper, we describe a patient with metastatic breast cancer showing signs of increased toxicity following capecitabine therapy. The DPD enzyme activity analysis revealed a partial deficiency. The study of the most frequent polymorphisms of the *DPYD* gene suggested a wild-type genotype but indicated a novel variant c.1903A>G (p.Asn635Asp), not previously described, proximal to the splice donor site of exon 14. After excluding the potential pathogenic feature of the newly-identified variant, we performed cDNA sequencing of the entire *DPYD* coding sequence. This analysis identified the variants c.85T>C and c.496A>G, which were previously described as pivotal components of the haplotype associated with decreased enzyme activity and suggested that both variant alleles are related to DPD deficiency. The clinical case findings described in this study emphasize the importance of performing complete genetic analysis of the *DPYD* gene in order to identify rare and low frequency variants potentially responsible for toxic reactions to fluoropyrimidine treatment.

## Background

Fluoropyrimidines, as fluorouracil, capecitabine and tegafur, are chemotherapeutic agents commonly used in the treatment of many solid tumors, including gastrointestinal, head and neck, and breast cancers (1). Up to 90% of the administered dose is physiologically inactivated in the liver by dihydropyrimidine dehydrogenase (DPD), an enzyme involved in the catabolism of the drug (2).

The *DPYD* gene (OMIM ^*^ 612779) which extends for 4,399 nucleotides and includes 23 coding exons on chromosome 1p22, usually presents a number of polymorphisms, transmitted in an autosomal recessive manner, which may result in a partial or absolute enzyme deficiency associated with an increased risk of toxicity (3, 4). Carriers of some of these allelic variants, even if heterozygous, are exposed to a high risk of developing severe toxicity such as neutropenia, nausea, vomiting, diarrhea, stomatitis, mucositis, hand-foot syndrome, and peripheral neuropathy with sometimes even fatal outcomes following fluoropyrimidine-based chemotherapy (3–5).

Among around 450 missense *DPYD* single-nucleotide polymorphisms (SNPs) reported in NCBI dbSNP (6) to date, only approximately twenty of them acquire a functional significance. Four of these variants are considered to be of clinical relevance for recognized effects on the protein, their identified toxic effects, and for their population frequency (7, 8): c.1905G >A (rs3918290, also IVS14 +1G >A), c.1679T >G (rs55886062), c.2846A>T (rs67376798) and c.1129–5923C>G (rs75017182, HapB3). This last variant is in linkage disequilibrium with the synonymous variant c.1236G> A (rs56038477) in Europeans (7) and is often employed during mutational screening analysis.

Therefore, the main scientific consortia and working groups in this field suggest adoption of analysis of *DPYD* mutations and suggest a reduction in fluoropyrimidine doses, or the adoption of an alternate drug in case of heterozygosity or homozygosity, respectively (4, 7, 8). However, it has been suggested that other *DPYD* polymorphisms could contribute to the variability in therapeutic responses, while several of the more common *DPYD* variants to date show conflicting evidence regarding their effects (7). Genotyping methods appear to be a more reliable approach to identify patients at risk of serious adverse reactions, compared to pharmacokinetic analyses and evaluations of enzymatic activity (9). Thus, development of an extensive screening method is a suitable tool for validating the potential pathogenicity of some variants, as well as for the proper management of cancer patients undergoing fluoropyrimidine treatment (10).

## Case Report

An 81 year-old woman was admitted to our oncology unit (Military Training Hospital Bégin, Unit of Oncology, Paris, France) for the management of grade 3 diarrhea due to fluoropyrimidine-related toxicity. She suffered from an invasive ductal carcinoma of the left breast (Elston-Ellis grade 3, RH^+^ HER2-) diagnosed in 2007. Medical management was initially based on surgery, radiation therapy and chemotherapy (adriamycine and cyclophosphamide). Hormonal therapy (anastrozole then exemestane) was performed during the following 5 years (2008–2012), and then she entered a regular clinical and radiological follow-up program. As bone and hepatic metastases were diagnosed in 2016 and 2017, respectively, hormonal therapy (exemestane) and chemotherapy (paclitaxel) were reintroduced. In May 2018, given evidence of CNS progression with multiple cerebral metastases, capecitabine (1,500 mg twice a day) was administered. The patient presented signs of major toxicity requiring urgent hospitalization in our department 20 days after capecitabine treatment, and the main symptoms included diarrhea (grade 3) and asthenia (grade 3). She was admitted to our department for close monitoring, with intensive fluid and nutritional support.

Biological investigations performed at admission revealed hematological toxicity with grade 4 neutropenia (absolute neutrophil count: 0.31 × 10^9^/L; reference interval, 1.5–4 × 10^9^/L) and grade 4 thrombocytopenia (platelet count: 35 × 10^9^/L; reference interval, 150–300 × 10^9^/L). The consequences of the severe diarrhea resulted in low blood levels of potassium (2.8 mmol/L; reference interval, 3.5–4.5 mmol/L), phosphate (0.4 mmol/L: reference interval, 0.81–1.45 mmol/L) and magnesium (0.63 mmol/L; reference interval, 0.7–1.05 mmol/L). Moreover, prothrombin time was increased (prothrombin ratio: 41 %; reference interval, >70 %) with a mild elevation of liver transaminase levels (ALT: 60 UI/L; reference interval, <33 UI/L and AST: 106; reference interval, < 32 UI/L) and a marked hypoalbuminemia (albumin level: 22.3 g/L, reference interval, 35–52 g/L). Stool cultures failed to detect bacterial pathogens, including *C. difficile* strains.

Capecitabine treatment was suspended upon the patient's admission to our service. The clinical evolution was slowly favorable: no diarrhea was observed after 72 h, and cell blood counts were normalized in 1 month.

**Figure 1:**
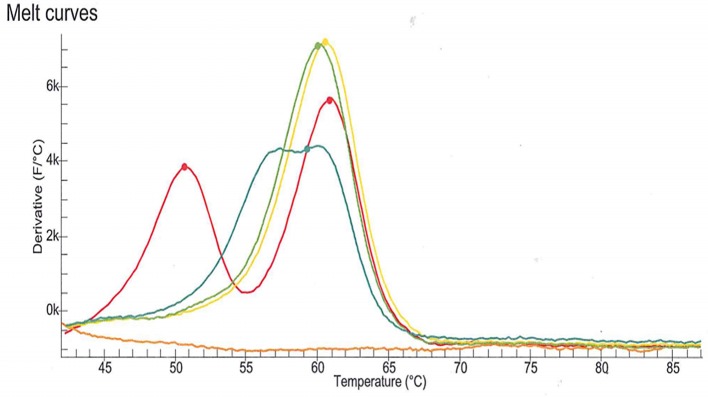
Melting curve relative to rs3918290 (c.1905+1G>A) genotyping using LAMP Human DPD deficiency kit (LaCAR MDx Technologies, Lièges, Belgium). The melting curve observed with the patient sample (blue line) is clearly different from those associated with the wild-type genotype (green and yellow lines) and the heterozygous genotype (red line). This atypical profile of the melting curve suggested the presence of an uncommon SNP.

DPD deficiency was suspected as a potential explanation for the severe toxicity following the first cycle of treatment with capecitabine. DPD phenotype assessment was performed by measurements of plasma uracil (U) and dihydrouracil (UH2) using an LC-MS/MS method (11). Analysis revealed a partial DPD deficiency according to the established criteria (U: 40.4 ng/mL, deficiency cut-off > 16 ng/mL and/or UH2/U ratio: 5.0, deficiency cut-off < 6) (12). The patient signed written informed consent for genotyping and related data for scientific research. The four *DPYD* SNPs most commonly observed in the Indo-European population were genotyped according to the current recommendations of the Clinical Pharmacogenetics Implementation Consortium (7). The SNPs (c.1905+1G>A, rs3918290; c.2846A>T, rs67376798; c.1679T>G; rs55886062 and c.1236G>A, rs56038477) were genotyped using LAMP Human DPD deficiency kit (LaCAR MDx Technologies, Lièges, Belgium). None of the four variant were detected. However, an atypical profile of the melting curve relative to rs3918290 genotyping was observed (Figure 1). As the presence of an uncommon SNP was suspected, sequencing of *DPYD* exon 14 was performed which revealed that the patient harbored a SNP in a heterozygous state: c.1903A>G (p.Asn635Asp) (chromosome position 1:97915617, A/G). To the best of our knowledge, this is a novel variant and is not identified in any of the specific Ensembl, dbSNP, ExAC, Alfred, HGMD or LOVD databases.

In order to evaluate the impact of the proximity of this variant to the intron 14 mRNA splice donor site, we used the most popular *in silico* tools to predict potential functional alterations involving splicing sites. SIFT (Scale-Invariant Feature Transform, http://sift.bii.a-star.edu.sg/, accessed 28 September 2018) and MutationTaster (http://www.mutationtaster.org/, accessed 28 September 2018) predictions suggested a deleterious effect for this variant, with scores of 0.002 and 23, respectively, while PolyPhen-2 prediction (http://genetics.bwh.harvard.edu/pph2/index.shtml, accessed 28 September 2018) indicated a benign effect, with a confidence score of 0.371. The PROVEAN (Protein Variation Effect Analyzer—http://provean.jcvi.org/index.php, accessed 28 September 2018) tool indicated a neutral prediction with a score of −1.770, while the Human Splicing Finder (htt://www.umd.be/HSF3/index.html, accessed 28 September 2018) tool referred to an exonic splicing enhancer (ESE) mutation “Alteration of an exonic ESE site. Potential alteration of splicing.” Furthermore, the SwissModel web tools (http://swissmodel.expasy.org/, accessed 28 September 2018) indicated that the missense variant did not affect the final structure of the protein.

**Figure 2:**
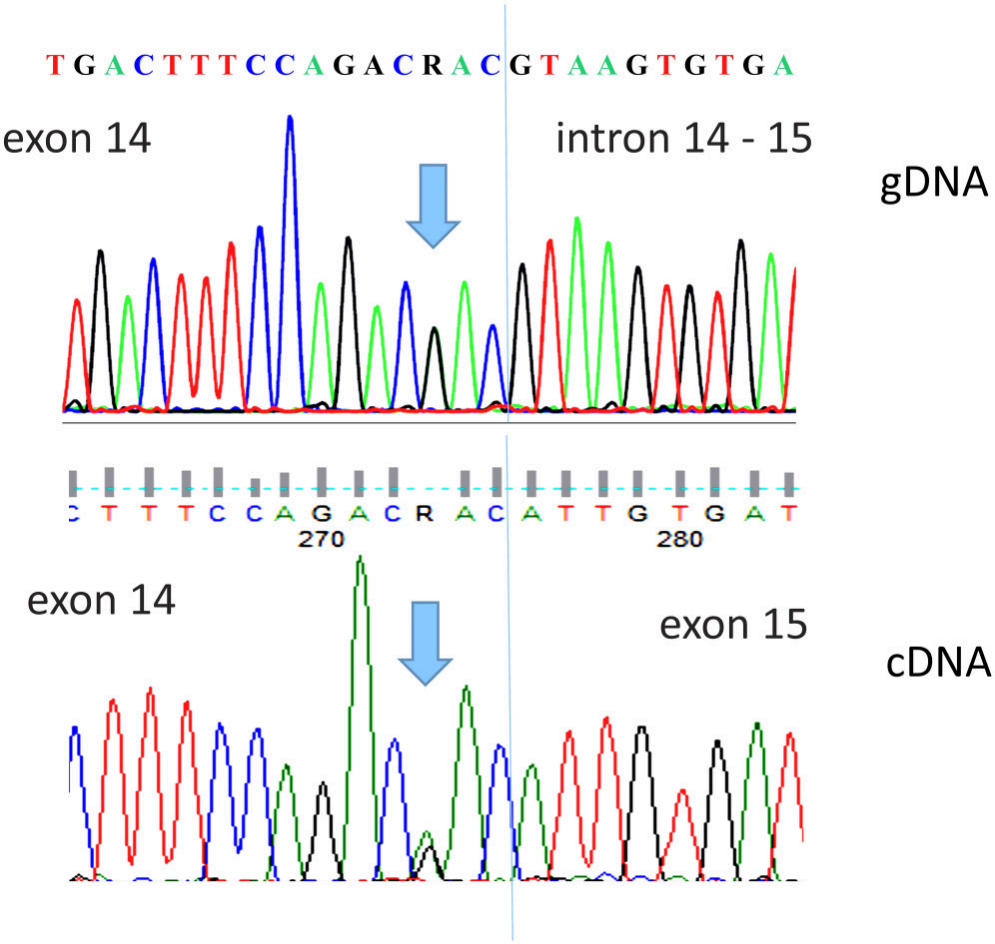
Direct sequence analysis of the DPYD c.1903A>G, p.Asn635Asp variant detected in our patient. The upper panel shows the gDNA sequence of exon 14 and intron–exon border. The lower panel shows the patient cDNA sequence, performed with primers spanning exons 13 and 15, demonstrating the integrity of exon 14.

Therefore, in order to characterize the potential pathogenic features of the identified variant, a peripheral blood sample stored in PAXgene Blood RNA Tube (Qiagen, Hilden, Germany) was sent to the Oncogenomic Research Center (University of Bari, Italy) for further molecular analysis. Total RNA was extracted from whole blood using the PAXgene Blood RNA Kit (PreAnalytiX GmbH, Hombrechtikon, Switzerland) and reverse transcribed into cDNA with an iScript cDNA Synthesis Kit (BioRad, Hercules, CA, USA). Using a pair of primers spanning exons 13 and 15 of *DPYD*, the cDNA was amplified and sequenced using a 3500 Genetic Analyzer (Applied Biosystems, 3500 Genetic Analyzer (Applied Biosystems, Foster City, CA, USA). Sequence analysis performed on the cDNA tract including exons 13–15 did not indicate exon 14 skipping (Figure 2).

The cDNA was then used to perform real-time quantitative PCR assays using the iTaq Universal SYBR Green Supermix (BioRad) in the Step One Plus instrument (Applied Biosystems). The mRNA levels were measured using 3 replicates per sample, with the comparative threshold cycle (Ct) method using glyceraldehyde-3-phosphate dehydrogenase (*GAPDH*) and beta-actin (ACTB) as endogenous controls, and with related values calculated by ΔCt. As controls, equal amounts of RNA extracted from whole blood of 3 subjects, previously genotyped and identified as wild-type for variants in the *DPYD* gene, were used. *DPYD* mRNA levels in the patient carrying the variant were reduced by an average of 45% compared to those observed in healthy subjects expressing the wild-type *DPYD* gene. A potential limitation in this analysis is that it is difficult to normalize across patients in samples containing multiple cell types that have different expression levels of house keeping genes, but normalizing with either a structural gene and a metabolic gene gave similar results, suggesting the effective reduction of gene expression in the patient.

As a subsequent analytical step, 5 pairs of primers were designed to perform sequencing of a 3296 bp cDNA segment, including the 3078 bp coding sequence of the *DPYD* gene (Table 1).

**Table 1:** Primers used in the DPYD cDNA amplification and sequencing (RefSeq: NM_000110, Transcript ID: ENST00000370192.7).

**Included region**	**Code sequence 5**^**′**^**-3**^**′**^		**TM**	**Size (bp)**
5′UTR-35–916	cDNA1 fw	TTGTCACTGGCAGACTCG	55	951
	cDNA1 rev	ATGTATAAAACCCCTGGTCC		
827–1946	cDNA2 fw	TACAAAGCTGCTTTCATTGG	56	1,139
	EX 13-15 rev	TTGGCAAGTTCCGTCCAG		
1605–2291	EX 13-15 fw	TAGAAATGGCCGGATTGAAG	58	686
	cDNA3 rev	TCCATATGTAGTTCGCTTTGC		
2209–2875	cDNA4 fw	ACTGTCTCAGGTCTGATGGG	58	676
	A2846T rev	TCATGTAGCATTTACCACAGTTGA		
2781–3′UTR+182	A2846T fw	AAGCACTGCAGTACCTTGGAA	59	482
	cDNA5 rev	TTGAATGGTCATTGACATGAGAC		

*TM, Melting Temperature; bp, base pair*.

Analysis of the results allowed the identification of the variants c.85T>C (Cys29Arg) and c.496A>G (Met166Val) recognized as the reference SNP (refSNP) Cluster Report rs1801265 and rs2297595, described with clinical significance of “*Pathogenic*” and “*With drug-response allele*” respectively (http://www.ncbi.nlm.nih.gov/snp/ accessed 28 September 2018).

The re-introduction of capecitabine at lower doses (1,000 mg twice a day) was associated with recurrence of adverse effects (grade 3 diarrhea and thrombocytopenia—platelet count: 88 × 10^9^/L) and treatment was stopped. Following this, new therapies were adopted (Palbociclib and Fulvestrant) until the patient died 3 weeks later.

## Discussion

Here, we describe the diagnostic procedure involving a breast cancer patient exhibiting fluoropyrimidine-related toxicity and subjected to *DPYD* mutational analysis. At first, during the molecular screening carried out to evaluate the variant IVS14 + 1G> A, a never previously described variant, c.1903A> G, was identified at the last codon of *DPYD* exon 14, encoding the amino acid substitution p.Asn635Asp. Therefore, to assess its potential pathogenic role, we used several Web tools to identify any possible deleterious effects on the protein. The prediction of its potential pathogenicity by several websites led us to perform both qualitative and quantitative mRNA analyses, which in all instances excluded any involvement of the splicing process, but confirmed a reduction in mRNA abundance.

At the same time, we also performed a complete mutational analysis of the *DPYD* gene, which revealed the presence of two other rare variants.

The c.496A>G (Met166Val) transition is referred to as “Damaging” by SIFT (score: 0.004), PolyPhen-2 (score: 1.00) and PROVEAN (score: −3.50). Analysis of the DPYD protein crystalline structure showed that this mutation has the ability to modify the integrity of the dimeric complex of DPYD, resulting in an alteration of a hydrophobic conserved three-dimensional environment determined by the interaction of amino acids L840, W849, Q852, V162, and I168 (13). A study conducted on 89 patients diagnosed with breast, gastroesophageal and colorectal cancer showed a significant G allele association with the phenotype of enhanced toxicity of grade III/IV (1). Other studies did not show a significant correlation between the presence of the variant and 5-FU toxicity (14, 15) and one study even demonstrated that the M166V variant exhibited significantly higher enzyme activity of approximately 120% compared to that of wild-type *DPYD* (16).

To date the c.85T>C (Cys29Arg) variant is considered neutral/tolerated by the SIFT algorithms (score: 0.19), PolyPhen-2 (score: 0.00) and PROVEAN (score: 0.08). The first *in vitro* studies performed on the c.85 T>C variant by analysis of expression in *Escherichia coli* resulted in a C29R inactive mutant DPD protein without residual enzymatic activity (17), while subsequent studies have shown a significant increase in functionality, exhibiting 13% higher activity than wild-type (18). Ocular side effects manifesting in the course of 5-FU-based chemotherapy have also been described in a subject carrying this mutation (19).

More recently the two mutations were included in a panel of 10 *DPYD* genetic variants for fluoropyrimidine-related adverse events (FAEs) retrospectively evaluated in the TOSCA (Three or six colon adjuvant) clinical trial (5). The study included 508 patients with surgically resected, stage III and high-risk stage II colorectal cancer, treated with 3 or 6 months of either FOLFOX-4 or XELOX adjuvant chemotherapy. The rs2297595 variant was one of the mutations that showed a shorter time-to-toxicity (TTT) with a 6.6 and 1.2 month TTT in the heterozygous and homozygous states, respectively. The deleterious effect of this variant is explained by the authors on the basis of the methionine-valine exchange at a site crucial for enzyme function and which is highly conserved through evolution (20). On the contrary, the same study did not show any association regarding the rs1801265 variant (5). Finally, a *DPYD* multi-SNP analysis performed to investigate the genotype-phenotype correlation involving 5-FU metabolism showed that the presence of minor alleles of the SNPs rs1801265 (C) and rs2297595 (G) determining the formation of a haplotype (Hap4, estimated frequency 8% in the Western population), is associated with a marked reduction in the values of 5-FU degradation rate (5-FUDR) (21).

## Conclusion

Here, we describe a diagnostic itinerary in a patient with metastatic breast cancer presenting symptoms of toxicity to capecitabine. In the first instance, during a mutational analysis of the four clearly pathogenic variants suggested by the main current guidelines, we identified a novel mutation at the terminal portion of exon 14. Despite the prediction of its pathogenicity by several computational algorithms, mRNA analysis allowed us to exclude a potential deleterious effect for this variant. However, the detection of reduced mRNA levels and the partial DPYD protein deficiency led us to extend the mutational analysis across the coding region of the entire *DPYD* gene. Numerous reports suggest today that the discovery of rare or novel mutations in the *DPYD* gene may account for an appreciable percentage of fluoropyrimidine toxicity in chemotherapy patients (22). Our analytical approach, based on the mutational analysis of the entire transcript of the DPYD gene in our patient, suggests that the rare variants identified may most likely be responsible for a reduction in mRNA levels, as also suggested by the previous scientific literature (1, 5, 17, 19, 20).

We believe that the solution adopted here, which provides a complete sequence analysis of the mRNA through the use of only 5 amplification reactions, provides a rapid and reliable method to identify rare sequence variants and splice site alterations (22). This can be an effective, inexpensive solution and is also achievable in most diagnostic centers and, in agreement with recent specific studies that identify an ideal screening for the identification of *DPYD* variants as “feasible and convenient, exceeding screening costs” (9, 23). Therefore, we hope that our experience can help to update specific databases and could be clinically relevant to address diagnostic paths useful for identifying fluoropyrimidine-related toxicity.

## Ethics Statement

The authors state that they have obtained appropriate institutional review board approval with written informed consent from the subject. Informed consent was obtained from the patient in order to publish this case report. The subject gave written informed consent in accordance with the Declaration of Helsinki.

## Author Contributions

RP, HD, and FS participated in the study's design and coordination, performed acquisition of data, and drafted the manuscript. DL and HD participated in molecular biology tests, data interpretation, and revised the manuscript. ALR and SC were resident in charge of patients during treatment and revised the manuscript. All authors read and approved the final manuscript.

### Conflict of Interest Statement

The authors declare that the research was conducted in the absence of any commercial or financial relationships that could be construed as a potential conflict of interest.
